# The Hallmarks of Aneuploidy in Cancer and Congenital Syndromes

**DOI:** 10.1146/annurev-genom-111723-103557

**Published:** 2025-05-07

**Authors:** Pan Cheng, Karan Singh, Roger H. Reeves, Teresa Davoli

**Affiliations:** 1 Institute for Systems Genetics and Department of Biochemistry and Molecular Pharmacology, New York University School of Medicine, New York, NY, USA;; 2 Department of Physiology, Johns Hopkins University School of Medicine, Baltimore, Maryland, USA

**Keywords:** aneuploidy, hallmarks, cancer, congenital aneuploidy, Down syndrome, chromosomal instability

## Abstract

Aneuploidy, characterized by the gain or loss of chromosomes, plays a critical role in both cancer and congenital aneuploidy syndromes. For any aneuploidy, we can distinguish between its general effects and its chromosome-specific effects. General effects refer to the common cellular stresses induced by aneuploidy, such as impaired proliferation, proteotoxic stress, and altered metabolism, which occur regardless of the specific chromosome involved and profoundly impact cellular and organismal functions. These generalized stresses often hinder cell fitness but can also, under certain conditions, contribute to cancer progression. In contrast, chromosome-specific effects arise from the altered dosage of particular genes on the gained or lost chromosome. These effects vary depending on the chromosome involved and can provide specific fitness effects in cancer cells or distinct developmental phenotypes in congenital aneuploidies like Down syndrome. Understanding the interplay between these two levels of effects is crucial for deciphering the outcomes of aneuploidy. This review synthesizes current knowledge and discusses future directions for unraveling the hallmarks of aneuploidy.

## 1. INTRODUCTION

### 1.1. General and Chromosome-Specific Effects of Aneuploidy

Animal species package their genomes in different numbers of chromosomes, ranging from a single chromosome in jack jumper ants to 225 chromosomes in the blue butterfly. Despite this interspecies variability, chromosome number is strictly maintained within each species. In fact, the presence of chromosome gains and losses, known as aneuploidy, is generally either lethal or severely detrimental to organismal fitness. Here, we review how chromosome number is faithfully maintained and what happens when aneuploidy occurs across cells and organisms.

Aneuploidy is defined as the presence of gains or losses of whole chromosomes or chromosome arms in the genome; whole-genome doubling, copy number alterations smaller than a chromosome arm, and structural variants are not the focus of this review (see the sidebar titled Chromosomal Gains, Losses, and Whole-Genome Duplication). In humans, with rare exceptions such as Down syndrome (DS), aneuploidy is incompatible with embryonic development (76); it is also uncommon at the cellular level, being virtually absent in healthy tissues (99). However, aneuploidy is exceptionally frequent in human cancers, occurring in more than 50% of hematopoietic tumors and more than 90% of solid tumors (20). Key questions about the roles of aneuploidy in both cancer and development relate to, first, how chromosomal gains and losses affect cellular physiology and state, and second, how this process influences cancer progression and developmental phenotypes.

**Figure 1:**
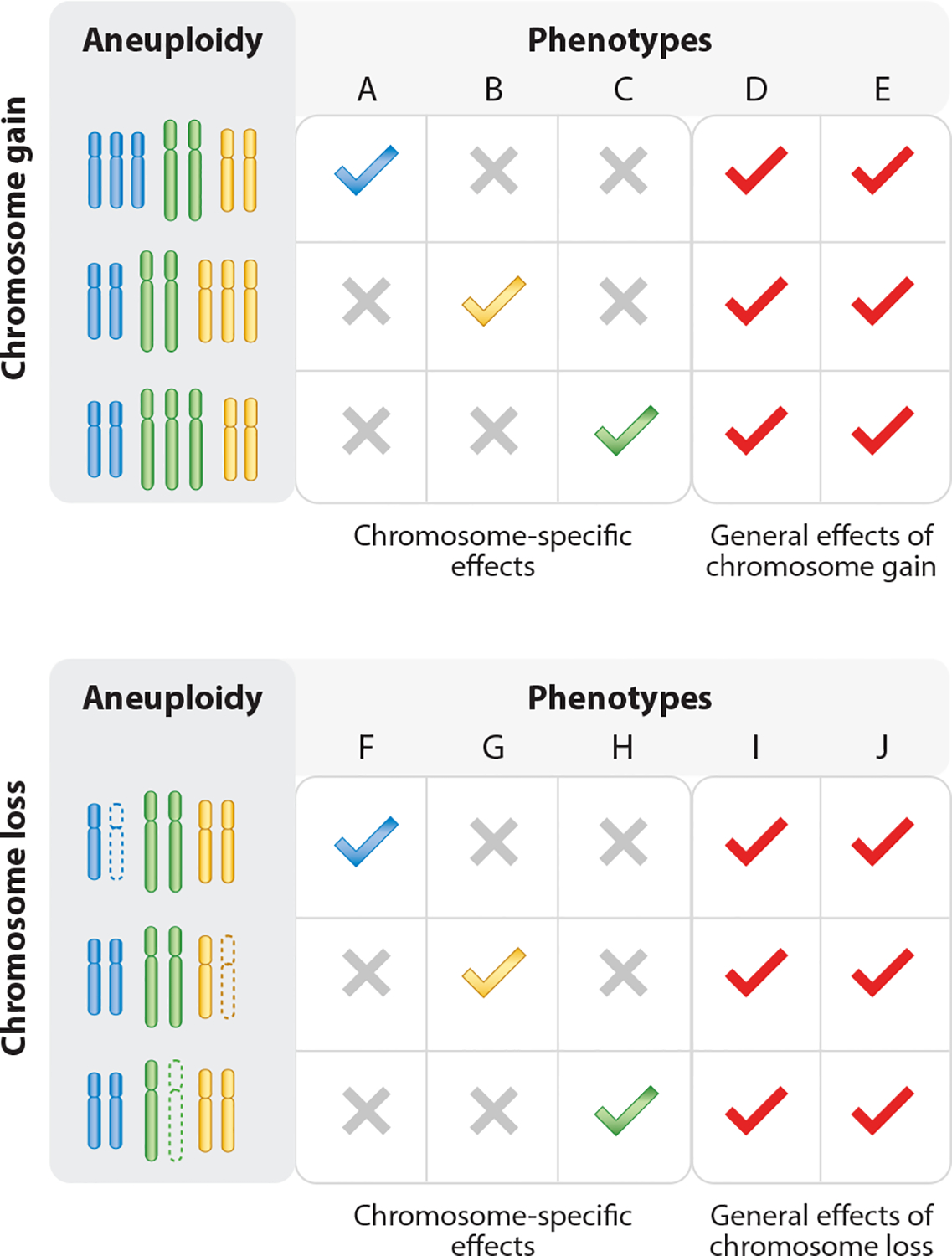
Representation of the general and chromosome-specific effects of aneuploidy. General effects are consequences of aneuploidy (either chromosome gain or loss) regardless of the specific chromosome that is gained or lost. Chromosome-specific effects are consequences that are unique to the gain or loss of specific chromosomes.

**Figure 2:**
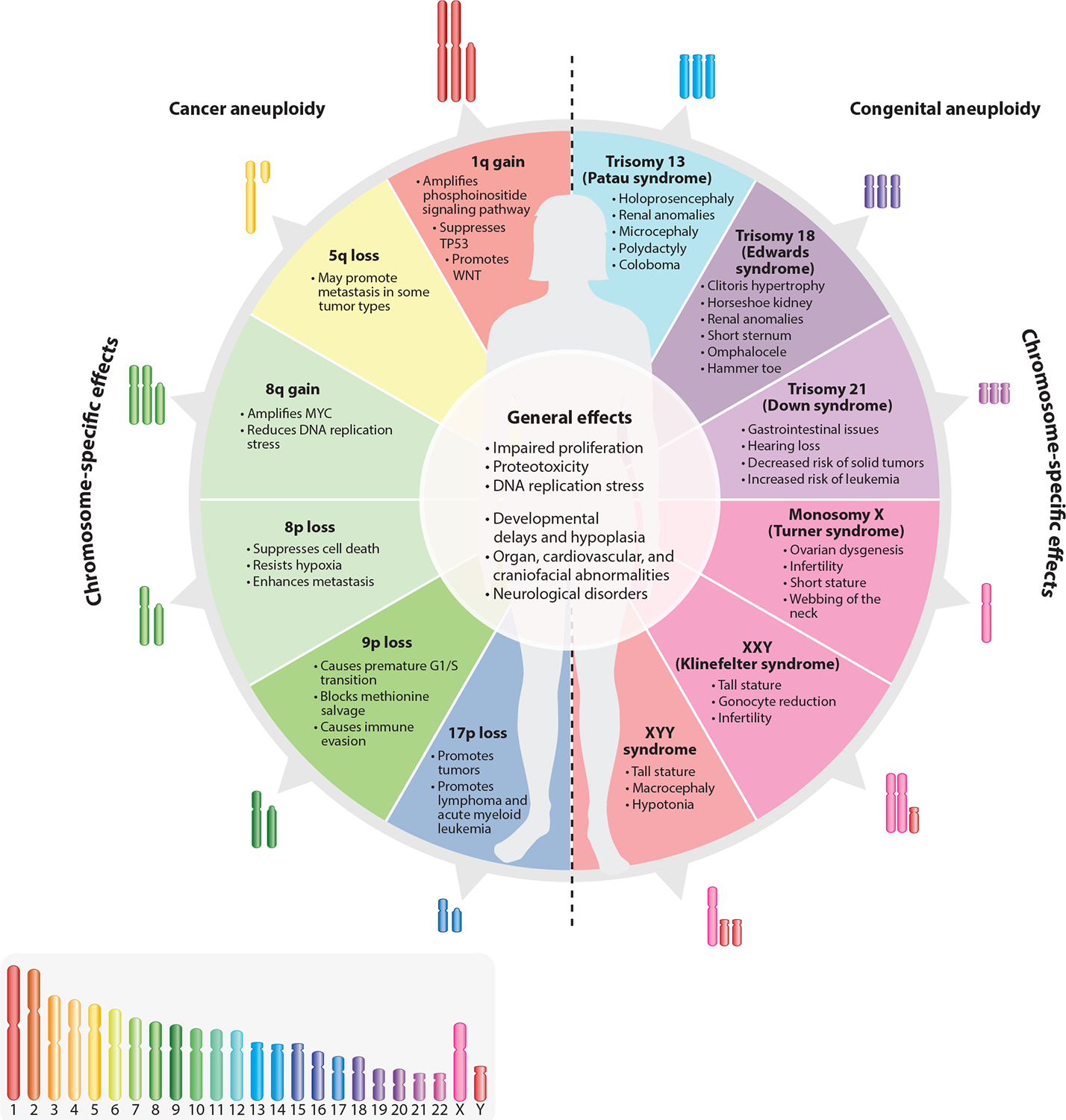
Overview of the main general and chromosome-specific effects of cancer aneuploidy (*left*) and congenital aneuploidy (*right*). Representative chromosomes are shown.

For virtually any given aneuploidy, we can distinguish between its general effects (i.e., consequences of aneuploidy independent of the specific chromosome gained or lost, common to virtually all aneuploidies) and chromosome-specific effects (i.e., consequences dependent on the specific chromosome gained or lost) (Figure 1). After summarizing the most common causes of aneuploidy, we review both the general and chromosome-specific effects of aneuploidy in cancer and congenital contexts (Figure 2). While cancer and congenital aneuploidies differ in that they affect cancer cell behavior and organismal phenotypes, respectively, in both cases we can recognize effects that are common among different aneuploidies and effects that are specific to the altered chromosome. Well-documented general effects in both contexts include proteotoxic stress, impaired cellular growth, and physiology. Additionally, there are key differences between the two contexts that we will highlight. For instance, in cancer, aneuploidy typically occurs in tumor cells that lack critical tumor suppressor genes such as *TP53* and are surrounded by genomically intact stromal and immune cells. In contrast, in congenital syndromes, aneuploidy occurs in *TP53*-intact cells from most or all body tissues, leading to wide-ranging and tissue-specific consequences for development and physiology.

### 1.2. The Interplay Between Aneuploidy and Chromosomal Instability

Aneuploidy arises through chromosomal instability (CIN) during mitosis or meiosis. While aneuploidy and CIN are often discussed together due to their close relationship, CIN is distinct from aneuploidy. CIN refers to the rate at which cells acquire structural or numerical alterations of chromosomes (see the sidebar titled Chromosomal Instability and Aneuploidy). CIN generates aneuploid cells, and aneuploidy can also promote CIN in some circumstances (62). However, it is crucial to note that aneuploid cells are not necessarily chromosomally unstable (6). For instance, the colorectal carcinoma cell line HCT116 (containing chromosome 5 gain) and cells from DS individuals maintain stable aneuploidy without ongoing CIN. This review focuses primarily on aneuploidy, except for specific instances where aneuploidy and CIN are so intertwined that it has not been possible so far to distinguish them; for a comprehensive review of CIN, we direct readers to Reference 4.

## 2. CAUSES OF CANCER AND CONGENITAL ANEUPLOIDY

**Figure 3:**
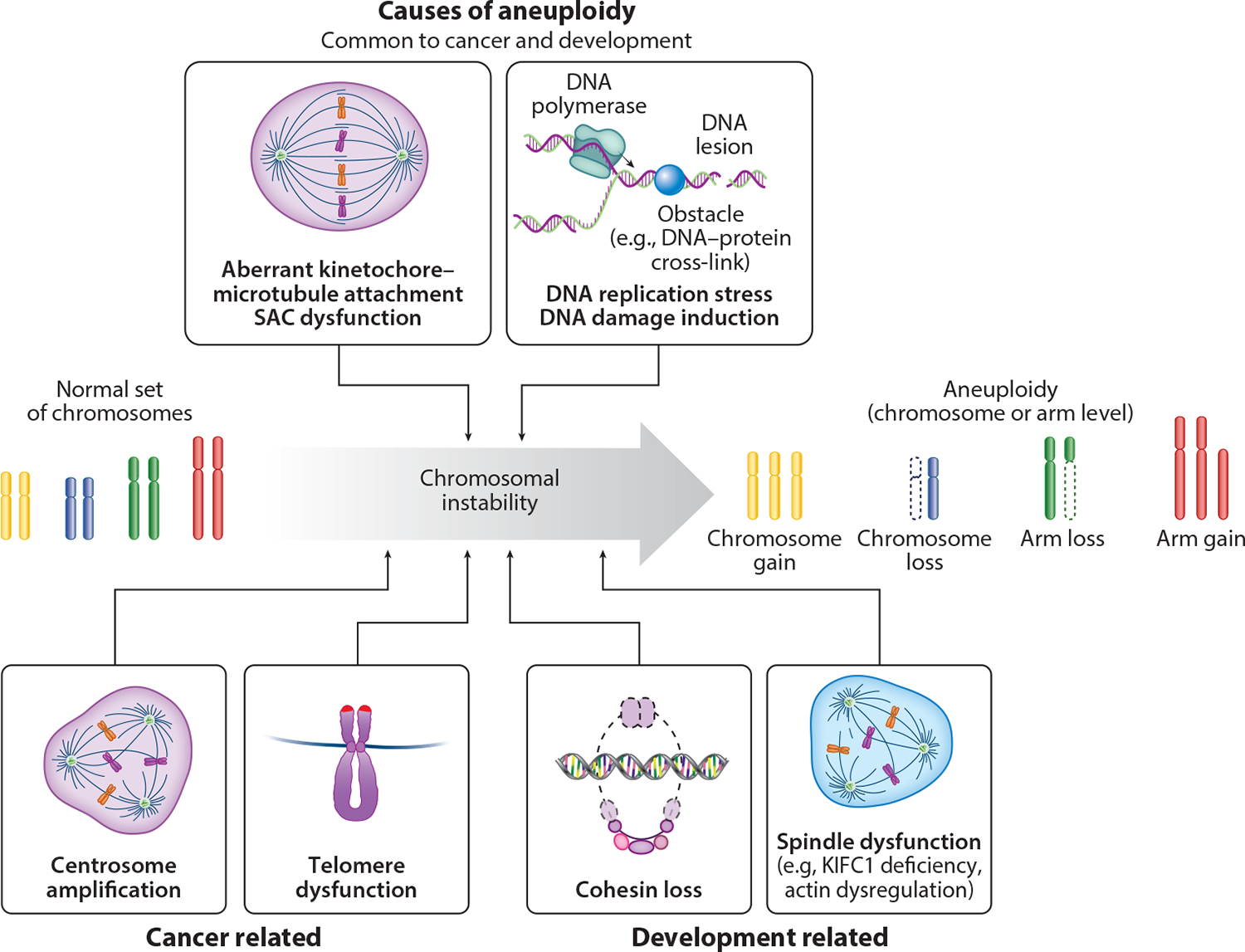
Main causes of aneuploidy in cancer and congenital syndromes. Causes that are common and specific to each of these contexts are shown. Among the common causes of aneuploidy, aberrant kinetochore–microtubule attachment, SAC dysfunction, DNA replication stress, and DNA damage induction are depicted. Among the context-specific ones, for cancer aneuploidy, centrosome amplification and telomere dysfunction are shown, and for developmental aneuploidy, cohesin loss and spindle dysfunction are shown. Abbreviation: SAC, spindle assembly checkpoint.

The accurate segregation of chromosomes during mitosis and meiosis is orchestrated by the intricate spindle apparatus and sophisticated cell cycle control mechanisms. Errors in this process result in aneuploidy, a hallmark of cancer cells and a common occurrence in the maturation of aged oocytes and derivation of human embryos (35, 66, 123). Given that the mechanisms underlying these errors have been extensively reviewed elsewhere (13), we only summarize the primary causes (Figure 3; Table 1).

A predominant source of chromosome segregation errors in both development and cancer contexts is the formation of aberrant kinetochore–microtubule attachments, categorized as monotelic (only one of the two sister kinetochores of a chromosome is attached to microtubules from a spindle pole, while the other remains unattached), syntelic (both sister kinetochores of a chromosome are attached to microtubules from the same spindle pole), or merotelic (a single kinetochore is attached to microtubules from both spindle poles). Unattached kinetochores or insufficient tension between sister kinetochores, resulting from monotelic or syntelic attachments, triggers the spindle assembly checkpoint (SAC). This crucial surveillance mechanism halts cell cycle progression into anaphase until errors are corrected (126). A compromised SAC has been observed in female meiosis, leading to elevated prevalence of aneuploidy (197). Intriguingly, aneuploid cancer cells typically exhibit a robust SAC (66, 191). Unlike monotelic and syntelic attachments, merotelic attachments evade SAC detection and are considered more detrimental, contributing significantly to chromosome missegregation in cancer (188). Notably, only a small proportion of persistent merotelic attachments in anaphase lead to chromosome missegregation, suggesting the existence of additional surveillance and correction mechanisms in anaphase for chromosome attachment errors that satisfy the SAC (122).

DNA replication stress (DRS) is another crucial factor that contributes to aneuploidy in both development and cancer (30). DRS can arise from stalled or abnormally accelerated replication forks and from collisions between transcription and replication machineries (164). This stress can result in incomplete replication or unresolved homologous-recombination repair intermediates, persisting into mitosis as ultrafine DNA bridges between sister chromatids (33). Unresolved ultra-fine DNA bridges may lead to chromosome breakage or lagging chromosomes, thereby inducing aneuploidy (34). Intriguingly, DNA synthesis during mitosis protects aneuploid cells from further DRS-induced genome instability (62, 131).

In cancer, centrosome amplification and telomere dysfunction represent other causes of chromosome segregation errors. Cells harboring supernumerary centrosomes may undergo multipolar division, yielding three or more daughter cells with substantial aneuploidy, which are generally unviable. Alternatively, additional centrosomes tend to cluster into two groups to support bipolar cell divisions, albeit still with an increased incidence of merotelic attachments and chromosome missegregation (59). In human cancer—especially in early-stage tumors—telomere dysfunction arises from telomere shortening. This results from the rapid cellular proliferation in the absence of telomerase activity (135). Dysfunctional telomeres, which cells perceive and thus repair as double-strand breaks, cause end-to-end chromosome fusions that, in turn, result in a variety of chromosomal aberrations, including structural rearrangements and aneuploidy (104, 135).

In oocyte maturation in aging females, cohesin loss has been implicated as a key driver of aneuploidy (38). Cohesin, a ring-shaped multiprotein complex, maintains sister chromatid cohesion, facilitating proper pairing and orientation of homologous chromosomes and accurate segregation of sister chromatids. In aged oocytes, cohesin levels drop below the required levels, resulting in random segregation of homologous chromosomes or premature sister chromatid segregation (109). Beyond cohesin loss, decreased spindle-associated F-actin may further contribute to destabilize sister chromatid associations, exacerbating premature chromatid separation (54). Another spindle component, the molecular motor KIFC1, is deficient in human oocytes compared with those of other mammals, rendering spindles in human oocytes unstable and susceptible to chromosome missegregation (175).

## 3. HALLMARKS OF CELLULAR ANEUPLOIDY AND ITS RELEVANCE TO CANCER

### 3.1. General Aneuploidy Effects

**Figure 4:**
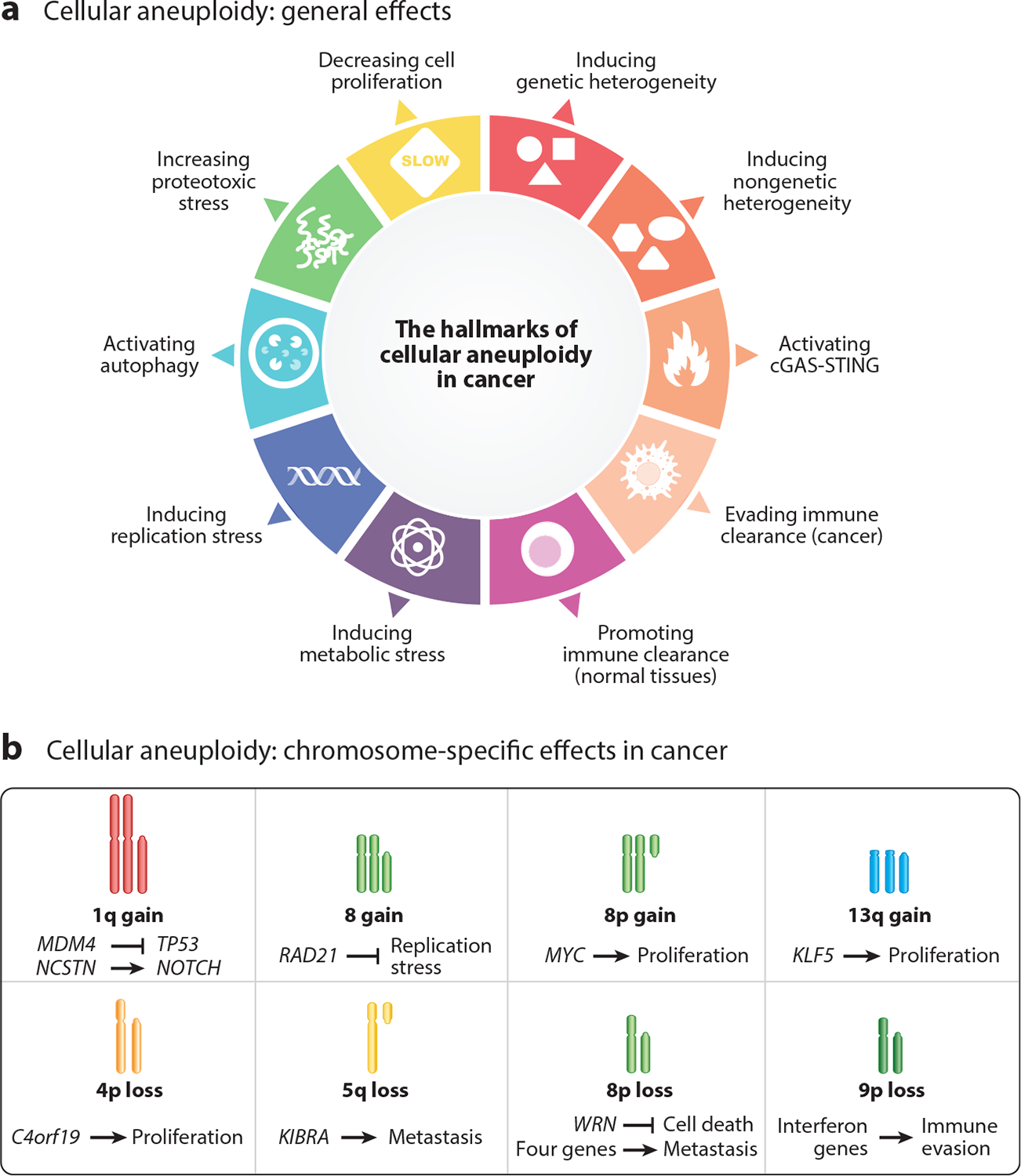
Hallmarks of cellular aneuploidy and its relevance to cancer. (*a*) Aneuploidy hallmarks are derived from the general aneuploidy effects on cell physiology. In one case (interaction with the immune system), we distinguish between the effects of aneuploidy in normal cells and in cancer cells. (*b*) Chromosome-specific aneuploidy hallmarks in cancer; for each specific chromosome gain or loss, the figure shows the gene(s) on the altered chromosome implicated in the phenotype(s). Note that there is no correspondence between the colors used for the hallmarks in panel *a* and the colors used for the chromosomes in panel *b*.

Here, we describe the general aneuploidy effects at the cellular level, which are relevant not only for the effect of aneuploidy in tumorigenesis (Figure 4a) but also for the effect of aneuploidy in congenital syndromes (see Section 4). We also discuss how certain general aneuploidy effects may promote tumorigenesis in certain contexts, e.g., in the absence of a functional TP53 pathway.

#### 3.1.1. Aneuploidy-associated cellular stresses.

In normal (noncancerous) cells and tissues, aneuploidy disrupts various physiological cellular processes, including proteostasis, metabolism, and DNA replication, ultimately impairing cell proliferation. While these effects are discussed here primarily in the context of tumorigenesis, they are also relevant to congenital aneuploidy syndromes (see Section 4.2).

##### 3.1.1.1. Impact on proliferation.

Aneuploidy impairs proliferation of nontransformed immortalized human cells, mouse cells, and yeast (179, 195, 216). Compared with chromosome gain (trisomy or tetrasomy), which can hinder cell proliferation, chromosome loss (monosomy) usually causes cell death or cell cycle arrest due to haploinsufficiency of human genes (especially of ribosomal protein genes) (40). The decreased proliferation after chromosome gains is likely due to the cumulative effect of different cellular stresses; however, the precise molecular mechanism remains poorly defined, given the complex nature of the phenotype, which affects multiple cellular pathways simultaneously (125). Initially, aneuploidy was found to activate p53 and cause p53-dependent cell cycle arrest in G1, thus limiting the proliferation of aneuploid cells (108, 189). Subsequent studies revealed that p53 activation and cell cycle arrest are triggered primarily by DNA damage associated with aneuploidy rather than by aneuploidy itself (160, 177). Furthermore, the relationship between aneuploidy and p53 activation appears to be context dependent. In organotypic cultures—3D cell cultures that mimic the architecture and function of native tissues—derived from epithelial tissues of neural, intestinal, and mammary origins in mouse and human models, aneuploidy failed to activate p53 or arrest cell proliferation (134). This discrepancy may be attributed to varying tolerances of aneuploidy-induced cell stress in different experimental systems. Cells in organotypic cultures, which more closely mimic in vivo conditions, may be better equipped to resist aneuploidy-induced stress, thereby avoiding p53 activation. Importantly, p53 is not the sole mediator of proliferation defects in aneuploid cells. A study using Nalm6 pre-B lymphocyte cell lines demonstrated p53-independent cell cycle arrest following aneuploidy induction (134), indicating the existence of additional, unidentified mechanisms that limit the proliferation of aneuploid cells. These observations highlight the complexity of cellular responses to aneuploidy. Further research should focus on characterizing both p53-dependent and p53-independent factors that may impact the proliferation of aneuploid cells in more physiologically relevant environments (125).

##### 3.1.1.2. Proteotoxic stress.

Proteotoxic stress, a condition arising from the overloading of cellular systems responsible for maintaining protein homeostasis (proteostasis), is widely recognized as a primary general consequence of aneuploidy (41, 66). In normal cells, proteostasis encompasses the correct folding of proteins, balanced expression and sophisticated assembly of protein subunits into complexes, and refolding or removal of misfolded and aggregated proteins (81). Disruption of these processes significantly impairs cell fitness. The major guardians of cellular proteostasis include molecular chaperones, the ubiquitin–proteasome system, and the autophagosomal–lysosomal system (81). Recent studies have implicated mitochondria in the clearance of cytosolic protein aggregates (115, 154). Aneuploid cells consistently exhibit signs of proteotoxic stress, including impaired protein folding (50); increased protein aggregation (28, 140); upregulation of proteasomal, autophagic, and lysosomal genes; and activation of the unfolded protein response (90, 139, 161, 179) in both yeast and human models. Corroborating the presence of proteotoxic stress in aneuploid cells, enhanced proteasome-mediated protein degradation via loss of UBP6 improves survival of aneuploid yeast (194), and augmented protein folding via HSF1 overexpression increases tolerance in aneuploid human cells (50). Furthermore, aneuploid cells exhibit increased sensitivity to various proteostasis-disrupting agents. For instance, aneuploid yeast shows heightened susceptibility to the proteasome inhibitor MG132 (195), and aneuploid mouse and human cells may exhibit increased sensitivity to 17-AAG (an HSP90 protein folding inhibitor) and chloroquine (an autophagy inhibitor) (185). Human aneuploid cells are also sensitive to genetic disruption of proteasomal genes (90). These findings collectively underscore the critical role of proteostasis maintenance in aneuploid cell survival and highlight the potential vulnerability of aneuploid cells to proteotoxic stress-inducing agents.

Although proteotoxic stress reduces the fitness of laboratory-generated aneuploid *Saccharomyces cerevisiae* strains (140), natural aneuploid isolates exhibit enhanced tolerance to proteotoxic stress through protein compensation via increased protein turnover through ubiquitination and proteasomal degradation (133). Strong compensation for noncomplex proteins could not be explained by the previous model that proteotoxic stress results primarily from protein complex imbalances (28, 143, 179). Similar compensation for noncomplex proteins is observed in aneuploid cancers (37), implying that similar mechanisms may exist in human malignancies. These findings raise questions about protein compensation mechanisms in aneuploid cells and cell type–specific tolerance to aneuploidy-induced proteotoxic stress, particularly as recent research has revealed that the burden of aneuploidy extends beyond protein homeostasis—DNA copy number changes also increase transcriptional load, making aneuploid cells more dependent on RNA degradation pathways (90).

##### 3.1.1.3. DNA replication stress.

DRS is another general consequence of aneuploidy, forming a complex bidirectional relationship. While studies have shown that DRS can induce CIN and consequently aneuploidy, new emerging evidence has demonstrated that aneuploidy itself can also induce DRS. For example, RPE-1 cells with acute aneuploidy induced by reversine (an MPS1 inhibitor) exhibit reduced replication fork rates, increased replication fork stalling and reversal, and elevated levels of DRS markers such as RPA (62, 160). Similarly, trisomic and tetrasomic RPE-1 and HCT116 cells exhibit slower replication rates and increased sensitivity to the replication inhibitor aphidicolin (141). Beyond these global challenges, replication stress at specific genomic regions, such as highly repetitive telomeres, has also been observed in aneuploid human fibroblasts and murine hematopoietic stem cells (128).

To mitigate elevated DRS, aneuploid cells fire dormant replication origins through a DDK-dependent mechanism and complete replication of genomic loci through mitotic DNA synthesis (62). Nevertheless, the sources of replication stress in aneuploid cells remain unclear. Contributing factors can include scarcity of replication components, depleted or imbalanced deoxynucleotide triphosphate pools, deregulated origin firing, and increased replication fork barriers, such as single-stranded breaks, secondary structures, interstrand cross-links, base lesions, DNA–protein cross-links, and R-loops (164, 227). The decreased and imbalanced levels of MCM2–7 proteins reported in aneuploid human cell lines (141) may indicate that a scarcity of replication factors is a potential source of replication defects in aneuploid cells. The consequences of DRS are multifaceted. DRS can produce double-stranded breaks that can be toxic when they accumulate at high levels. Additionally, ATR can be activated to suppress origin firing, change genome-wide fork speed, and induce cell cycle arrests in S and G2/M phases. Lastly, underreplicated DNA can persist into mitosis, resulting in chromosome bridges and CIN (164). Consistent with elevated DRS, increased DNA damage and genome instability have been observed in aneuploid cells (41, 66, 91, 141, 168), but whether DRS causes these alterations remains to be determined.

##### 3.1.1.4. Metabolic stress.

Metabolic rewiring is another well-documented phenomenon associated with aneuploidy. In yeast, aneuploid strains disomic for chromosome 4 or chromosomes 11 and 16 exhibited more glucose uptake than haploid controls (195). In mammalian cells, aneuploid mouse embryonic fibroblasts trisomic for chromosomes 1, 13, 16, or 19 consistently displayed elevated glutamine consumption and increased production of ammonium and lactate, while glucose consumption and glutamate production varied in a karyotype-specific manner (216). These findings suggest a general alteration in energy production in aneuploid cells, although the specific changes can be karyotype dependent. Nevertheless, not all metabolic pathways are uniformly affected by aneuploidy. Most aneuploid yeast strains examined showed no significant changes in intracellular pools of amino acids or tricarboxylic acid (TCA) cycle intermediates (190).

A consistent metabolic alteration observed across aneuploid yeast, mouse, and human cells is the accumulation of sphingolipid metabolites. A study on the above aneuploid yeast strains revealed that metabolites of the sphingolipid family, including long-chain bases and ceramides, accumulated in most strains (87). This accumulation, driven by increased serine biosynthesis, was unique among lipid classes and has profound implications for cellular physiology. Intriguingly, the accumulation of long-chain bases improved the fitness of aneuploid cells by promoting anabolism and energy production, while ceramide accumulation decreased cellular fitness. Similar increases in ceramide levels were observed in aneuploid mouse embryonic fibroblasts and high-aneuploidy colorectal cancer cells, rendering aneuploid cells sensitive to interventions that further increased ceramide levels (186). Furthermore, aneuploidy induced nuclear membrane abnormalities in aneuploid yeast and human cells, which could be alleviated by increasing cellular long-chain base levels (88). These results establish a direct link between sphingolipid metabolism alterations and nuclear morphology in aneuploid cells, with significant implications for overall cell fitness. It is not surprising that metabolic changes can depend on specific karyotypes and cellular contexts. Further research should examine metabolic profiles using aneuploid models with sufficient diversity in karyotypes and cells of origin.

In conclusion, aneuploidy induces diverse cellular stresses that significantly impair cell fitness. The mechanisms by which these stresses emerge and are managed by aneuploid cells remain an active area of research. A critical avenue of investigation involves examining stress responses associated with both the initial occurrence of aneuploidy and the subsequent adaptive stages. This temporal resolution can provide insights into how aneuploid cells evolve to cope with these challenges over time (26, 210). Furthermore, comparing stress responses and adaptation mechanisms across different cells of origin offers an intriguing opportunity to identify both common and distinct patterns in aneuploidy tolerance. Such comparative studies could reveal cell type–specific vulnerabilities or resilience factors. Finally, understanding these aneuploidy-induced stresses opens new avenues for designing novel therapeutic strategies. For instance, aneuploid cells exhibit heightened sensitivity to further increases in proteotoxic stress, exacerbation of genome instability, modulation of specific metabolites like ceramide, or disruption of chromosome segregation (43, 62, 87, 185). Leveraging these unique vulnerabilities may make it possible to selectively target aneuploid cells in cancer.

#### 3.1.2. General aneuploidy effects fueling cancer.

While the general cellular effects of aneuploidy described in Section 3.1.1 are typically tumor-suppressive, as they reduce cell proliferation and overall fitness, cancer cells often exploit these effects to their advantage. This usually occurs in the presence of *TP53* inactivation, which is the somatic genetic event most strongly associated with aneuploidy across cancers. In this context, aneuploidy can promote tumorigenesis, for example, by enhancing intratumor heterogeneity and facilitating metastatic spread.

##### 3.1.2.1. Association with TP53 inactivation in cancer.

While aneuploidy can induce diverse cellular stresses that impair cell fitness, it remains unclear whether certain general aneuploidy effects can promote cancer. The interaction between aneuploidy and common mutations in oncogenes or tumor suppressor genes is crucial. Among tumor-associated mutations, those in *TP53* exhibit a particularly strong correlation with aneuploidy (46, 187). *TP53*, the most frequently mutated tumor suppressor gene in cancer, encodes a key transcription factor activated in response to various cellular stresses, such as DNA damage, oncogene activation, hypoxia, oxidative stress, and replication or translation stress. It regulates a broad spectrum of genes involved in crucial cellular functions, including DNA repair, apoptosis, cell cycle arrest, senescence, and metabolism (25). Beyond the loss of tumor-suppressive functions of wild-type *TP53*, certain *TP53* mutations can acquire gain-of-(abnormal)-function properties that further promote cancer (73). Emerging evidence suggests mutated *TP53* can equip cancer cells with the ability to cope with challenging conditions during tumorigenesis, including DNA damage, proteotoxic stress, and nutrient fluctuations—conditions that significantly overlap with aneuploidy-induced cellular stresses (124). This leads to a compelling hypothesis: *TP53* mutations may help cells tolerate and adapt to the cellular stresses induced by aneuploidy, potentially mitigating its negative impacts on cell fitness while preserving other aneuploidy effects that can facilitate cancer progression (discussed below). However, most aneuploid models used to study general aneuploidy effects have intact *TP53* (161, 179, 195, 210, 216), limiting our understanding of aneuploidy’s impact on cancer. Consequently, investigating the general effects of aneuploidy in *TP53*-mutant backgrounds is crucial to elucidate their roles in cancer initiation and progression, which may open intriguing directions for future research.

##### 3.1.2.2. Karyotypic and intratumor heterogeneity.

One of the most direct effects by which aneuploidy fuels cancer progression is the karyotypic heterogeneity that provides evolutionary and selective advantages to tumor cells. Two independent groups demonstrated these advantages by inducing transient CIN and consequent aneuploidy through inhibition of the SAC. These heterogeneous aneuploid populations exhibited enhanced chemotherapy resistance compared with diploid controls, with resistant populations eventually displaying specific karyotypes (89, 119). Karyotypic heterogeneity peaked following CIN induction but declined after exposure to chemotherapy agents, leading to the selection of resistant cells with specific karyotypes. These in vitro studies may reflect the process of how CIN promotes tumor progression in vivo. However, it should be noted that karyotypic heterogeneity is a direct consequence of CIN rather than aneuploidy per se. In contrast, homogeneous stable aneuploid cell populations would not exhibit such advantageous phenotypes.

##### 3.1.2.3. Epigenetic heterogeneity and adaptation to microenvironmental stresses.

Beyond genetic heterogeneity, aneuploidy can generate epigenetic heterogeneity. Cells sharing identical aneuploid karyotypes show heterogeneity in various phenotypes, such as cell cycle profiles and gene expression patterns, that are caused not by changes in DNA sequence but by variations in gene regulation. While the underlying molecular mechanism is poorly understood, epigenetic heterogeneity likely influences cancer evolution and drug resistance (18, 166). Furthermore, in certain contexts, aneuploidy-induced cellular stresses may paradoxically promote cancer progression. The slow proliferation and G1 phase delays characteristic of aneuploid cells render them resistant to the chemotherapeutic agents cisplatin and paclitaxel, which induce cell death primarily during S and M phases, respectively (148). These findings align with emerging evidence suggesting that slow-proliferating dormant cancer cells can persist after treatment, causing tumor relapse (162). These mechanisms may explain the observation that human aneuploid colon cancer cells trisomic for chromosomes 7 or 13 exhibited improved proliferation relative to diploid controls in nonstandard culture conditions such as serum starvation, hypoxia, and 5-fluorouracil exposure (155).

##### 3.1.2.4. Metastasis and immune evasion.

Immunosurveillance can also be affected by aneuploidy. Analyses of human tumors revealed an inverse correlation between aneuploidy levels and CD8^+^ T cell infiltration (46, 187), suggesting that aneuploidy may promote immune evasion. This decreased tumor immunity is closely associated with the unfolded protein response, a general aneuploidy effect related to aneuploidy-induced proteotoxic stress (223). Specifically, an enhanced unfolded protein response in aneuploid cells leads to the release of factors that polarize human macrophages toward an immune-suppressive phenotype and suppression of T cell activation. A similar mechanism underlies the observation that CIN tumors exhibit higher metastatic potential (107), which is associated with the activation of the cGAS–STING pathway. cGAS, a cytosolic DNA sensor, detects missegregated chromosomes in ruptured micronuclei and produces cyclic GMP–AMP signals that activate the downstream STING pathway. This activation results in the production of proinflammatory cytokines (121), typically linked to tumor suppression through induction of senescence, apoptosis, and immune clearance (51, 232). Paradoxically, one study found that chronic activation of cGAS–STING can activate immune-suppressive ER stress signaling, promoting immune evasion (107). Whether and (if so) how CIN-associated micronuclei induce cGAS–STING activation are still matters of discussion in the field (184).

Furthermore, how cGAS–STING affects cancer progression at different stages and in different contexts remains an important area of ongoing research. Notably, some in vitro studies have demonstrated contrasting effects of aneuploidy on immunosurveillance. Two studies on *TP53*-intact aneuploid cells induced by MPS1 inhibitors found that complex aneuploidy activated cell cycle arrest and senescence, resulting in the activation of the senescence-associated secretory phenotype, nuclear factor kappa B (NF-κB), and proinflammatory signals. Activation of these pathways promoted the clearance of aneuploid cells by natural killer cells (160, 208). However, whether this natural killer cell–mediated clearance is specific to aneuploid cells with intact *TP53* and senescence and its role in tumorigenesis and tissue homeostasis remain to be determined. Altogether, aneuploidy may promote tumor cells to escape from immune surveillance, although this effect may depend on aneuploidy degrees, model systems employed (e.g., MPS1 inhibitor induced versus genetically driven aneuploidy/CIN), specific karyotypes, and the stage or context of cancer progression. More studies are needed to elucidate the relationships between aneuploidy and immune responses.

In conclusion, while previous studies have extensively documented the negative effects of aneuploidy on cellular fitness, the potential for aneuploidy to promote tumor progression through certain general aneuploidy effects remains an active area of investigation. Recent findings have illuminated several effects through which aneuploidy may fuel cancer progression, including increased karyotypic heterogeneity, slowed proliferation, and enhanced immune evasion. Further research, particularly involving tumor-like contexts, is needed to fully elucidate the impact of general aneuploidy effects on cancer development and progression. Of particular interest is the impact of *TP53* mutations, which may alter the cellular response to aneuploidy-induced stresses and potentially unmask protumorigenic effects.

### 3.2. Chromosome-Specific Aneuploidy Effects

This section describes the patterns of chromosome-specific and tissue-specific aneuploidy across cancers and their possible role in promoting tumorigenesis by altering the dosage of cancer driver genes (20) (Figure 4b). We delve into the impact of aneuploidy on gene expression and highlight the crucial role of dosage-sensitive genes in shaping cancer aneuploidy patterns. Furthermore, we examine recent experimental studies that directly investigate the selective advantages conferred by specific chromosomal alterations for cancer progression.

#### 3.2.1. Chromosome-specific and tumor type–specific aneuploidy patterns across cancers.

Comprehensive cancer genome analyses reveal that aneuploidy typically affects more than 25% of the genome, with whole-genome doubling events correlating with higher aneuploidy levels (187, 212). Aneuploidy prevalence varies across cancer types, from low in thyroid carcinoma and acute myeloid leukemia to high in ovarian and lung cancers (187).

Across all cancers, chromosomes 8p and 17p are most frequently deleted (33% and 35%), while 8q is most commonly gained (33%) (187). Cancer-specific patterns exist; for example, lung adenocarcinoma and squamous cell carcinoma share 5p gain but differ in other alterations (102, 187). Analysis of The Cancer Genome Atlas samples revealed distinct aneuploidy clusters: gastrointestinal tumors show gains in 8q, 13q, and chromosome 20; gynecological and certain epithelial tumors share 1q gain; neural crest–derived and mesoderm-derived cancers feature chromosome 7 gain; and squamous cancers exhibit 3p loss and 3q gain (187). These patterns likely reflect tissue-specific selective advantages.

#### 3.2.2. Selection drives emergence of chromosome-specific aneuploidy in cancer.

From a theoretical standpoint, chromosome-specific patterns of aneuploidy might arise because certain chromosomes tend to missegregate more than others (frequency of occurrence) or because chromosomes missegregate at similar rates but selective pressure acts on them during tumorigenesis, leading to the emergence of events advantageous for the tumor cells (selection). These mechanisms are not mutually exclusive. Recent research has unveiled a mechanical bias in chromosome segregation errors, correlating with chromosomal position within the interphase nucleus. Peripheral chromosomes situated behind spindle poles exhibit the highest error rates due to the greater distances they must traverse to reach the metaphase plate (97). Beyond chromosomal location, the abundance of centromere components of individual chromosomes, such as CENP-B and kinetochore protein levels, and how much different chromosomes are susceptible to cohesion fatigue after mitotic delay impact the rate of chromosome missegregation (52, 221). Chromosome size shows a modest correlation with missegregation frequency; this association likely results from a combination of factors, including nuclear position and centromere features.

The varying missegregation rate among human chromosomes described above does not explain the frequencies of aneuploidy observed in human tumors. Rather, chromosome-specific aneuploidy is viewed predominantly as a consequence of selection during cancer evolution, wherein alterations to specific chromosomes confer contextual fitness advantages (209). Several mechanisms have been proposed to explain these advantages, including a model relating to the density of oncogene and tumor suppressor genes within chromosomes. This model posits that chromosomes dense in oncogenes tend to be gained, while those rich in tumor suppressor genes are often lost. In other words, the cumulative haploinsufficiency of tumor suppressor genes and triplosensitivity of oncogenes (chromosome gains and losses often affect only one copy of the chromosomes) drive chromosome-level and arm-level aneuploidy patterns in cancer genomes (47). This paradigm effectively explains the tissue specificity of aneuploidy patterns observed in different cancers, as the relative importance of these genes varies across tissues, resulting in distinct degrees of selective advantages for certain chromosomal alterations in different cancers (157). Chromosomal loci whose copy number alterations induce fitness advantages or disadvantages in cancers were further located using a new computational approach termed BISCUT (breakpoint identification of significant cancer undiscovered targets) (169). This method identified 193 genomic loci under apparent positive or negative selection, which were significantly enriched for known tumor suppressor genes and oncogenes (169).

Unlike compensated genes, haploinsufficient and triplosensitive genes are sensitive to DNA dosage changes. The loss of a single copy of a haploinsufficient gene or the gain of a single copy of a triplosensitive gene can alter their normal functions, leading to abnormal phenotypes. Therefore, aneuploidy provides an excellent platform to identify dosage-sensitive genes, where the association between copy number variations and disease phenotypes can be evaluated. A genome-wide RNA interference proliferation screen identified STOP and GO genes, which negatively and positively affect cell proliferation, respectively. Intriguingly, hemizygous (affecting one of the two alleles) recurring focal deletions in cancer genomes were enriched for STOP genes and depleted of GO genes, suggesting that their haploinsufficiency promotes tumorigenic phenotypes. Moreover, the densities of tumor suppressor genes and oncogenes, identified via the Tumor Suppressor and Oncogene (TUSON) Explorer algorithm, predict the frequency of losses and gains of chromosomes (and chromosome arms), respectively (47, 176). These findings suggest that the cumulative haploinsufficiency of tumor suppressor genes and triplosensitivity of oncogenes drive chromosome-level and arm-level aneuploidy patterns in cancer genomes.

Intriguingly, aneuploidy patterns are also tumor type specific. These patterns have been linked to gene expression patterns of corresponding normal tissues. Chromosomes whose genes have higher or lower levels of gene expression (compared with those of other chromosomes) in normal tissues overlap with chromosomes gained or lost in corresponding cancers (142). This observation implies that specific aneuploidies may provide selective advantages by hard-wiring tissue-specific gene expression levels in aneuploid genomes.

Beyond altering the dosage of driver genes, specific aneuploidy patterns have also been associated with cancer mutation profiles as a buffering mechanism. This model proposes that chromosomal gains can increase the likelihood of preserving wild-type genes when mutations occur, thereby mitigating the deleterious effects of gene mutations (3). However, the relationship between chromosome losses and mutation patterns remains unclear in this model. Taken together, the selective advantages conferred by copy number alterations of specific chromosomes shape the aneuploidy profiles observed in cancer. These selective advantages depend on the tissue of origin, as different tissues involve distinct genes and pathways for optimal fitness, resulting in tissue-specific patterns of aneuploidy.

#### 3.2.3. Selective aneuploidies promote cancer cell-autonomous and non-cell-autonomous cancer phenotypes.

Experimental evidence directly examining the selective benefits of specific chromosomal alterations remains limited (Table 2). One major obstacle stems from the technical challenges in modeling chromosome-specific aneuploidy. Recent advancements in genetic tools, such as KaryoCreate (Karyotype CRISPR-Engineered Aneuploidy Technology) and ReDACT (Restoring Disomy in Aneuploid Cells Using CRISPR Targeting), offer promising solutions to overcome this hurdle (17, 27, 31, 56, 65, 187, 198, 200).

Experimental studies that have examined the selective advantages of aneuploidy can be broadly divided into three types based on their methods of generating aneuploidy models (Table 2). The first involves long-term evolution experiments in vitro or in vivo, with or without specific stress (2, 89, 98, 118, 120, 127, 199, 210). In these studies, cells are often induced to be chromosomally unstable before evolution, typically by inhibiting the SAC or deleting *TP53*. The resulting karyotypes of surviving cells with growth advantages are then assessed, and potential driver genes are identified and tested. When specific stresses are applied during evolution, the final karyotypes often contain aneuploidies for specific chromosomes that confer resistance to the applied stress (2, 89, 118, 120). For instance, long-term exposure to paclitaxel induces loss of chromosome 10, which in turn confers paclitaxel resistance (118), and prolonged culture in serum-free conditions promotes gain of chromosome 7 that provides a growth advantage in such conditions (120).

The second approach compares established cell lines or cancer cells from tumors with or without specific aneuploidies (85, 100, 180). Similar to the first method, these cells have undergone evolution during cell line establishment or tumor formation. However, a limitation of this method is the potential confounding effect of different genetic backgrounds between cancer cells.

The third approach leverages advancements in genome editing techniques. These methods enable the deletion of large chromosome segments to construct cells with arm or chromosome losses using CRISPR (17, 27, 56, 65, 98, 169, 187), transcription activator–like effector nucleases (TALENs) (31), or Cre-LoxP (116) or the generation of trisomy through micronuclear-mediated chromosome transfer (93, 136). These techniques have been used to examine the selective advantages of gains in chromosomes 1q, 8, and 13q, as well as losses in 3p, 8p, 9p, 11q, and 18q in various cell types (17, 27, 31, 56, 65, 86, 93, 136, 169, 187) (Table 2). These studies identified some driver genes responsible for the selective advantages, such as the increased expression of MDM4 due to 1q gain, which may block TP53 activation (65, 85). Another example is the association between chromosome 9p loss and immune evasion, as well as resistance to immune checkpoint therapy (46, 215, 229). These immune-associated advantages may be attributed to copy number losses in the interferon (IFN) gene cluster (17). Notably, the selective advantage may result from dosage changes across multiple genes, rather than changes in a single gene (47, 86, 225), further complicating the identification of underlying driver genes. This underscores the need for continued refinement of experimental techniques and analytical approaches to fully unravel the selective advantages of changes in chromosome copy number.

#### 3.2.4. Dosage effects of aneuploidy at the RNA and protein levels: open questions.

The mechanisms underlying chromosome-specific selective advantages in cancers, as discussed above, fundamentally rely on the premise that DNA dosage changes can alter the expression and function of potential driver genes on aneuploid chromosomes. Our understanding of this complex relationship was dramatically enhanced by recent technological advancements in proteogenomic analyses of tumors, including DNA sequencing, RNA sequencing, and quantitative proteomic profiling. The phenomenon where gene expression changes are less than expected based on gene dosage alterations is termed gene compensation. Studies on aneuploid yeast and human cells have demonstrated that increased chromosome copy numbers generally lead to proportionally increased RNA levels for most genes on aneuploid autosomes (37, 49, 165, 179), suggesting that gene compensation for autosomes is minimal at the RNA level. However, gene compensation has been widely observed at the protein level for genes on aneuploid autosomes. It was initially thought that such compensation occurs primarily in protein complexes such as ribosomes (49, 179), but recent studies have revealed that both complex genes and noncomplex genes exhibit strong protein compensation (37, 133). These observations raise important questions for efforts to identify key genes driving chromosome-specific aneuploidy, as the expression of such genes should theoretically not be compensated. A recent study examining gene compensation in aneuploid tumors found that a chromosome gain event increased the expression of average oncogenes by only 12%, and a loss event decreased the expression of average tumor suppressor genes by a mere 8% (165). For instance, some oncogenes (e.g., *MYC*, *MTOR*, and *ERBB4*) and tumor suppressor genes (e.g., *BRCA1*, *CDKN1A*, and *CDH1*) exhibited strong protein compensation. These findings underscore the importance of carefully evaluating expression change when assessing the physiological impact of DNA dosage alterations.

These results are apparently inconsistent with the theory postulating cumulative gene dosage of oncogenes and tumor suppressors as one of the main driving forces of aneuploidy selection during tumorigenesis. To help resolve this discrepancy, these analyses might benefit from considering tumor type–specific driver genes, tumor stage, *TP53* status, and exact copy number alterations (e.g., whether the gains or losses occur in a diploid or tetraploid background genome). Intratumor (and intra–cell line) heterogeneity and CIN may also affect these analyses and cannot be fully accounted for in bulk tumor analyses. Most importantly, the cumulative effect of small dosage changes may be able to impact cellular phenotypes, and this is an important area of research (see Section 5).

Beyond the *cis* effects of DNA dosage changes on gene expression, aneuploidy may also impact the expression of genes on unaltered chromosomes through *trans* effects. One potential mechanism for this phenomenon involves the altered expression of transcriptional factors, DNA methylation enzymes, or histone modifiers on aneuploid chromosomes, which in turn affects the expression of target genes on unaltered chromosomes (117, 132).

## 4. HALLMARKS OF CONGENITAL ANEUPLOIDY

### 4.1. Congenital Aneuploidies in Mammals

In mammals, aneuploidy often leads to severe developmental abnormalities, frequently causing embryonic lethality (75). Embryos with any monosomy are nonviable, with the only exception being X chromosome monosomy (Turner syndrome) (24, 167). The lethality of autosomal monosomies likely stems from the dosage reduction of haploinsufficient genes and the unmasking of remaining recessive deleterious mutations (39). However, X chromosome monosomy appears to have a less severe impact on gene expression due to an existing compensation mechanism. This mechanism, known as X inactivation, transcriptionally silences one X chromosome in female cells, mitigating the effects of X chromosome loss (39). Trisomies are also generally incompatible with embryonic development; only three autosomal trisomies are viable in humans, and only one is viable in mice (68, 171). In both species, the viable autosomal trisomies involve chromosomes with the smallest numbers of genes, indicating that the cumulative effect of gene dosage changes decreases organismal fitness. Interestingly, studies of human embryos derived from spontaneous miscarriage and in vitro fertilization have shown that the frequency of aneuploidy across chromosomes in early development does not correlate with the frequency of aneuploidy at birth (i.e., almost all autosomal trisomies are observed in early development in at least 5% of embryos, while only three are compatible with life) (24, 74). These findings strongly indicate that the aneuploidy patterns compatible with development and life depend on the selection of the ones that can survive, rather than on different frequencies of occurrence.

The three autosomal trisomies compatible with human life are (*a*) trisomy 21 (DS), seen in ~1:700 live births; (*b*) trisomy 18 (Edwards syndrome), seen in ~1.3:10,000 live births; and (*c*) trisomy 13 (Patau syndrome), seen in ~0.85:10,000 live births (218). While Edwards and Patau syndromes lead to critically shortened life spans, with five-year survivals of 12% and 10%, respectively (130), DS individuals have a median life expectancy of 60 years (11). Other viable congenital aneuploidies involve the X chromosome. These include monosomy X (Turner syndrome), seen in ~1:2,000 live births (106), as well as the presence of extra copies of the X chromosome in Klinefelter syndrome (XXY) and triple X syndrome (XXX), both seen in ~1:1,000 live births (82). Individuals with sex chromosome trisomies generally have normal life spans (144).

**Figure 5:**
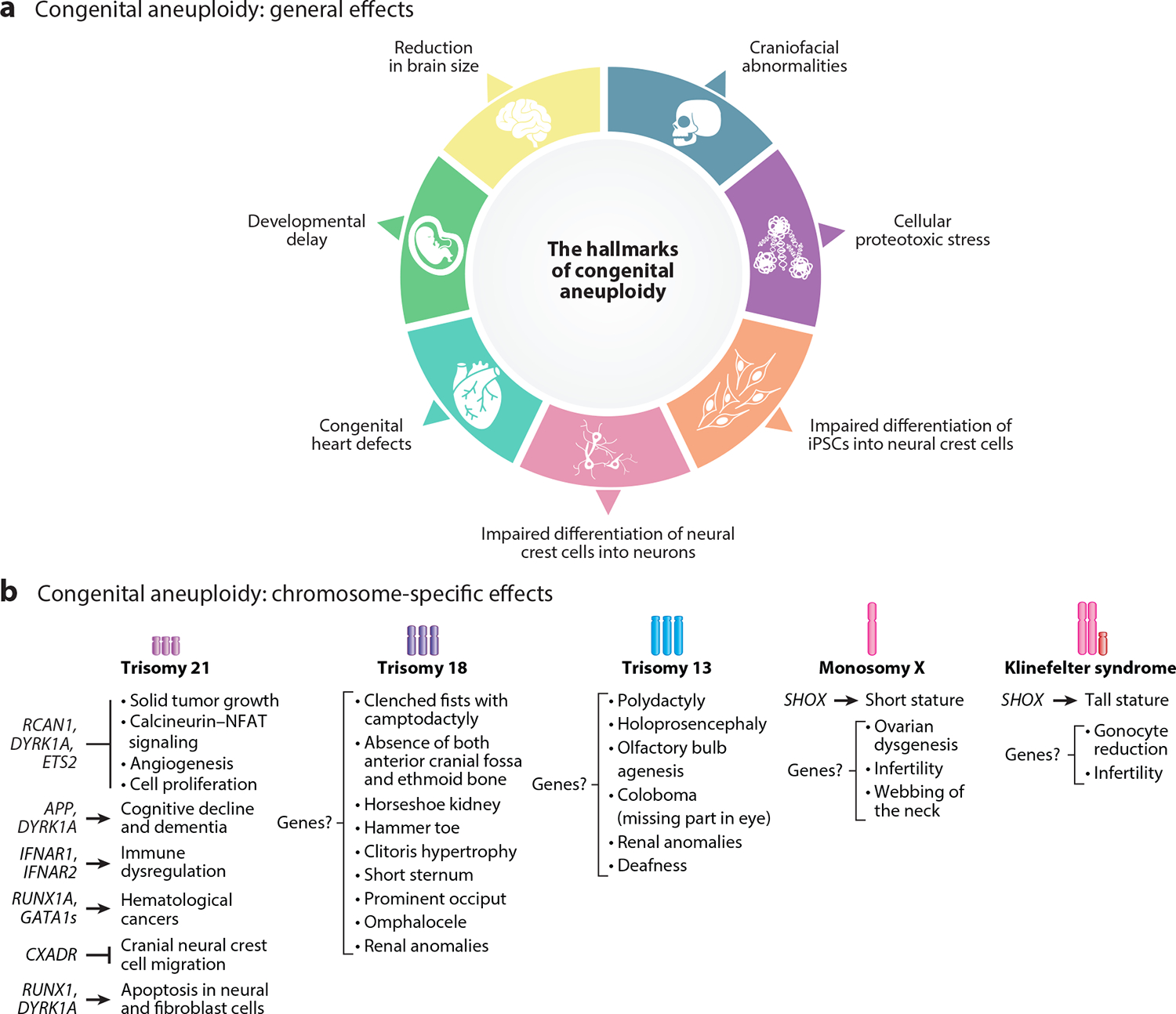
Hallmarks of congenital aneuploidy. (*a*) General aneuploidy hallmarks in congenital aneuploidy syndromes. (*b*) Chromosome-specific aneuploidy hallmarks in congenital syndromes; when known, for each specific chromosome gain or loss, the figure shows the gene(s) on the altered chromosome implicated in the phenotype(s). Note that there is no correspondence between the colors used for the hallmarks in panel *a* and the colors used for the chromosomes in panel *b*. Abbreviation: iPSC, induced pluripotent stem cell.

As in the case of cellular effects of cancer aneuploidies (Section 3), we can distinguish general and chromosome-specific effects of congenital aneuploidies at the organismal level. Below, we summarize the main types and possible causes of these two categories of effects (Figure 5).

### 4.2. General Effects of Congenital (Autosomal) Aneuploidies

Virtually all of the general effects of aneuploidy described above (Section 3 and Figure 4a) in the context of cancer also occur in congenital aneuploidies, including reduced proliferation and proteotoxic, metabolic, and replication stress. However, compared with the effects of cellular aneuploidy, the effects of aneuploidy at the organismal level are far more detrimental (Figure 5a).

First, hypoplasia (i.e., underdevelopment of an organ or tissue) and developmental delays (i.e., delayed achievement of development milestones) are common across all trisomies in humans and mice, including those surviving to birth (78, 207). Other species, including *Caenorhabditis elegans*, *Drosophila melanogaster*, and *Arabidopsis thaliana*, consistently exhibit aneuploidy-induced growth reduction (196). Given the limited mechanistic insights into this common organismal phenotype across various species, we propose several hypotheses based on the effects of aneuploidy in individual cells (Section 3). At the cellular level, unlike in cancer, organismal aneuploidy typically occurs in the presence of a functional TP53 pathway, potentially triggering senescence or cell death, which impedes development. At the organismal level, the sophisticated differentiation programs during development and organogenesis, involving highly precise spatiotemporal interactions among different cells, are more easily perturbed by the aneuploidy-associated stresses compared with the processes regulating individual somatic cells. Further studies are needed to better elucidate the mechanisms of aneuploidy-induced developmental delays at the organism level.

Second, organ abnormalities are a hallmark of human trisomies, manifesting with varying degrees of severity across different chromosomal disorders. Skeletal anomalies, particularly craniofacial abnormalities, and congenital heart defects are prevalent among human trisomies (207). These structural malformations are not limited to humans; trisomic mouse models also exhibit analogous phenotypes, such as craniofacial abnormalities and nuchal edema (19, 150). This interspecies concordance underscores the fundamental impact of aneuploidy on developmental processes, particularly in some sensitive parts, such as the skeletal and cardiovascular structures.

Third, neurological anomalies (e.g., learning and memory impairments or reduced cerebellar neuronal numbers) are also prevalent across different trisomies (207). Extensive in vitro and in vivo studies using induced pluripotent stem cells (iPSCs) derived from human cells with trisomy 21 and mouse models of DS suggest that the gain of chromosome 21 induces abnormal neural differentiation, alters the proportion of brain cells (e.g., excess GABAergic interneurons), impacts synaptic activity, and impairs recognition memory, among other neurological abnormalities (5, 16, 29, 32, 45, 61, 80, 158, 214, 224). However, similar comprehensive studies on other trisomies are currently lacking, precluding definitive conclusions about the universality of mechanisms discovered in trisomy 21 cells. Nevertheless, some hypothetical mechanisms may explain the common occurrence of neurological anomalies across different trisomies. Sterling et al. (178) demonstrated that aneuploid cortical cells undergo both p53-dependent and p53-independent apoptosis during brain development in a mouse model of mosaic aneuploidy, suggesting that cell death may be an underlying cause of aneuploidy-induced neurological anomalies. Additionally, proteotoxicity, a hallmark of aneuploidy discussed in Section 3, is closely associated with neurological disorders (156). The accumulation of misfolded proteins and disruption of protein homeostasis in aneuploid cells may contribute to the neurological deficits observed across various trisomies. Further research is needed to elucidate the shared and unique pathways by which different trisomies affect the nervous system.

All phenotypes discussed above highlight the impact of aneuploidy on cell differentiation, a factor often overlooked in the context of single well-differentiated cells and cancer aneuploidy. However, it may play a crucial role in congenital aneuploidy phenotypes. Studies examining human embryonic stem cells (hESCs) with various aneuploidies, derived either from spontaneous aneuploidization of hESCs in vitro or from human embryos, have revealed differentiation defects. For instance, spontaneously derived aneuploid hESC cultures exhibit impaired in vitro differentiation along different lineages, including neural lineage, extraembryonic endoderm, and placenta (21, 57, 111). Research on hESCs derived from diploid and aneuploid embryos (trisomies 13, 18, and 21) also indicates that all aneuploidies display abnormal expression of genes involved in differentiation toward several lineages, including the nervous system (23).

In conclusion, congenital aneuploidies have profound effects at the organismal level, leading to widespread developmental abnormalities. These include growth delays, organ malformations, and neurological deficits, which are observed across various species and trisomies. The impact on cell differentiation appears to be a critical factor in these phenotypes, highlighting the complex interplay between aneuploidy and developmental processes. Further research is needed to fully elucidate the mechanisms underlying these common effects of congenital aneuploidy on mammalian trisomies.

### 4.3. Chromosome-Specific Effects of Congenital Aneuploidies

Each of the congenital aneuploidies mentioned below has been extensively reviewed elsewhere (129, 159); here, we discuss phenotypes specific to different aneuploidies in humans that have been attributed to specific genes located on the altered chromosome (Figure 5b; Table 3).

#### 4.3.1. Trisomy 21 (Down syndrome).

Before discussing DS phenotypes, we briefly summarize the DS mouse models. Early attempts began in 1974 with the Ts16 mouse (67), which carried a full trisomy of mouse chromosome 16, but this model was inadequate due to its embryonic lethality. A key breakthrough came in the 1990s with the Ts65Dn mouse model (146). Ts65Dn is trisomic for a portion of chromosome 16 that is orthologous to human chromosome 21, carrying ~100 human chromosome 21 orthologs plus ~30 non–chromosome 21 orthologous protein-coding genes. This is a true trisomy with an extra freely segregating chromosome and was the standard for DS research for many years. Advances in chromosome engineering supported development of over 20 direct duplications of segments of mouse chromosomes 16, 17, and 10 (7). These models are useful for studying gene dosage effects. For example, studies showed that congenital heart defects in DS are due to at least two genes, including *DYRK1A* (102a).

In 2020, scientists introduced TcMAC21, a trans-chromosomic mouse model of DS that contains the human chromosome 21q arm (94). This humanized model replicates characteristics of human DS, including blood-related anomalies associated with increased leukemia susceptibility in DS individuals. Its comprehensive gene dosage representation makes it valuable for testing therapeutic interventions. More recently, a rat model, TcHSA21rat (95), has been described that contains a nearly complete copy of the entire human chromosome 21, including both the long and short arms. Compared with mice, rats support refined testing of many aspects of learning, memory, and development, plus metabolic and pharmaceutical testing.

##### 4.3.1.1. Neurological phenotypes.

DS is characterized by various neurological anomalies. Studies have shown that elevated expression of *OLIG2* (located on 21q22.11) in neural progenitor cells of the ventral forebrain can have significant developmental consequences. One study using DS iPSC–derived cerebral organoids and mice with chimeric aneuploid brains (generated by transplanting DS iPSC–derived neural progenitor cells into neonatal mouse brains) found that increased *OLIG2* expression led to an overabundance of GABAergic interneurons and impaired recognition memory in these chimeric models. In both models, *OLIG2* downregulation decreased the number of GABAergic interneurons and improvements in cognitive behavior (224). In another study, genetically deleting the extra copy of *Olig1*/*2* in Ts65Dn mice (Ts65n) reversed the overproduction of interneurons in the brain (32). Additionally, DS iPSC–derived cortical neurons show synaptic reduction in glutamatergic and GABAergic subtypes (214), and DS iPSCs generate fewer NR2F2 (also known as COUP-TFII)–positive progenitors with reduced proliferative capacity. This phenotype is likely due to diminished WNT signaling, as it is partially rescued by WNT signaling activation (57). The gene or genes on chromosome 21 responsible for WNT signaling deregulation are unknown.

Ts65Dn mice have a smaller cerebellum with fewer granule and Purkinje neurons, likely due to decreased response to Sonic hedgehog signaling (151, 214). Several studies point to *DYRK1A* (located on 21q22.13) as one of the main culprits of defective neuronal phenotypes. In one study involving teratoma formation in NOD-SCID mice, DS iPSCs displayed defective differentiation into neural progenitor cells and neurons (80). Increased dosage of *DYRK1A* likely contributes to this phenotype, as chemical or genetic inhibition of DYRK1A improves neurogenesis by enhancing cell proliferation and reducing apoptosis (80). Another study showed that overexpression of DYRK1A consistently alters neuronal differentiation and inhibits cell proliferation when it is overexpressed in neural progenitor cells (61). Finally, one study found that RUNX1 upregulation in DS IPSC–derived neural progenitor cells plays a role in disrupting neural differentiation. Using CRISPR-Cas9 to delete all RUNX1 isoforms helped reverse apoptosis in neural progenitor cells (70).

Individuals with DS have an increased risk of early-onset Alzheimer disease. Interestingly, the *APP* gene, which plays a significant role in Alzheimer disease pathology, is located on chromosome 21 (21q21.3). Duplication of either the *APP* locus alone or other genes on chromosome 21 can contribute to Alzheimer disease development, cognitive decline, and dementia (153, 217). The chromosome 21 gene *DYRK1A* is also involved in neurological development and may contribute to Alzheimer disease in individuals with DS (61, 173). Studies using mouse models have explored the effects of normalizing *Dyrk1a* dosage. In Ts65Dn mice, reducing the extra *Dyrk1a* copy by crossing Ts65Dn with *Dyrk1a*^+/−^ males rescued spatial working memory, reference memory, and contextual conditioning, as well as hippocampal long-term potentiation (61). Similarly, experiments crossing Dp1Yey with *Dyrk1a*^+^/^−^ mice or pharmacologically inhibiting brain DYRK1A corrected cognitive deficits (92, 138).

DS is strongly associated with a 100-fold increased risk of Hirschsprung disease, a congenital neurodevelopmental disorder characterized by a deficient enteric nervous system due to failure of rostro–caudal migration and proliferation of the enteric neural crest–derived cells in the developing gastrointestinal tract (9, 79). This leads to intestinal blockage and failure to pass meconium at birth. A significant number of Hirschsprung disease patients carry pathogenic alleles in *RET*, located on chromosome 10, and *EDNRB*, located on chromosome 13 (192). Both *RET* and *EDNRB* are essential for the development and differentiation of the enteric neural crest–derived cells (36, 206), with approximately 12% of isolated cases and 35–50% of familial cases carrying rare pathogenic alleles in the coding exons of RET (69, 193). Why individuals with DS have a higher risk of developing Hirschsprung disease remains unknown. Genetic factors likely play a key role, with the extra copy of chromosome 21 in DS possibly influencing Hirschsprung disease development. Another possibility is that secondary genetic modifiers on other chromosomes contribute to the increased risk.

##### 4.3.1.2. Cancer phenotypes.

In addition to neurological defects, individuals with DS exhibit a unique pattern of tumor type incidence: They are less likely to develop solid tumors, including breast, colon, and lung cancers, but more likely to develop hematological malignancies (71, 72). Both phenotypes have been attributed to specific genes located on chromosome 21 and their interactions with genes on other chromosomes. In acute megakaryoblastic leukemia, an interaction between *RUNX1* (on chromosome 21) and mutated *GATA1* (on the X chromosome) induces transcriptional dysregulation and inhibits megakaryocytic differentiation (213). Normally, the binding between the transcription factors GATA1 and RUNX1B/C is critical for the proper binding and regulation of megakaryocytic promoter regions (55). In DS-associated myeloid leukemia with *GATA1s* mutation, there is an imbalance in RUNX1 isoforms, with RUNX1A being overabundant relative to RUNX1C. This excess of RUNX1A blocks the binding of RUNX1C to the promoter site in the presence of mutant GATA1, thereby hampering the differentiation of stem cells into megakaryocytes and promoting myeloid leukemia (63). Restoring the balance between RUNX1A and RUNX1C reverses these effects both in vitro and in vivo (63).

Individuals with DS have a reduced risk of solid tumors in general and an extremely low risk of neuroblastoma (163). The decreased risk of tumors has been attributed in part to the overexpression of specific tumor suppressors located on chromosome 21. In the case of neuroblastoma, decreased risk has been attributed to overexpression of chromosome 21 genes such as *DYRK1A* and *RCAN1* (formerly known as *DSCR1*) (149, 170). Baek et al. (12) showed that posttransplant tumor models—Lewis lung carcinoma and B16F10 melanoma cells in Ts65n and transgenic *Rcan1* mice—exhibited inhibited tumor growth compared with wild-type mice; however, the reduction of tumor growth was more pronounced in Ts65n mice than in transgenic *Rcan1* mice. They further demonstrated that the deletion of one copy of *Rcan1* in Ts65n mice reversed the B16F10 tumor growth suppression phenotype at a modest level, suggesting that it acts as a tumor suppressor. Returning *Rcan1* to the normal two copies by deleting one copy of the gene in Ts65n mice (Ts65n-*Rcan1*^+/−^) does not completely restore the B16F10 tumor growth phenotype and microvessel density to wild-type levels, indicating that one or more additional genes on chromosome 21 are involved in the tumor growth suppression. Baek et al. (12) also increased the dosage of the *Dyrk1a* gene in endothelial cells derived from *Rcan1* transgenic mice and observed reduced VEGF-mediated endothelial cell proliferation. This was achieved by suppression of the VEGF–calcineurin–NFAT signaling. In other studies, increased dosage of RCAN1 and DYRK1A synergistically reduced NFAT signaling (10). By inhibiting calcineurin, RCAN1 reduces NFAT activity, leading to decreased angiogenesis and potentially limiting tumor growth (12). Furthermore, ETS2, a transcription factor involved in cell growth, differentiation, and apoptosis, appears to contribute to tumor suppression in DS (183). Transgenic overexpression of ETS2 at physiological levels in DS may enhance neuronal and fibroblast apoptosis and inhibit cell proliferation (219, 220).

##### 4.3.1.3. Other phenotypes.

Most individuals with trisomy 21 exhibit craniofacial skeletal abnormalities. Liu et al. (114) demonstrated that these craniofacial malformations are linked to defects in cranial neural crest migration. They showed that the *CXADR* gene, which encodes an adhesion protein and is located on chromosome 21, is more highly expressed in iPSCs derived from individuals with DS compared with disomy control lines. Downregulating the *CXADR* gene corrected the migration defects in cranial neural crest cells, suggesting that the *CXADR* gene plays a key role in cranial neural crest migration.

Chromosome 21 is notable for containing genes that encode four of the six known IFN receptors: *IFNAR1* and *IFNAR2*, which encode receptors for type I IFNs; *IFNGR2*, which is involved in type II IFN signaling; and *IL10RB*, which has a multifaceted role. IL10RB not only participates in type III IFN pathways but also acts as a receptor component for other cytokines, including IL10, IL22, and IL26 (182). As a result, the heightened IFN signaling contributes to an increased proinflammatory response (e.g., IL6, IL2, IL10, CXCL10, CCL2, IL22, and TNFa), which may help explain the chronic low-grade inflammation and immune dysregulation commonly observed in DS individuals (181, 182). This abnormal immune response may be a key factor in the higher susceptibility of DS individuals to certain autoimmune conditions (8).

#### 4.3.2. Trisomy 18 (Edwards syndrome).

Trisomy of the entire chromosome 18 is associated with 8% survival at one year; thus, most Edwards syndrome individuals have trisomy of only a portion of chromosome 18 or display mosaicism, where only a subset of cells carry the extra chromosome (222). Of the general aneuploidy effects mentioned above, craniofacial abnormalities are among the most prominent (147); reduced total body and brain weights are also common features (207). The specific features of Edwards syndrome are clenched fists with overlapping fingers, renal anomalies, short sternum, horseshoe kidney, hammer toe, hypertrophy of the clitoris, prominent occiput, and omphalocele (more common in trisomy 18 than in trisomies 13 and 21) (152). Edwards syndrome–derived iPSCs undergo spontaneous differentiation, during which they lose the extra chromosome 18, possibly due to initial mosaicism; this indicates selective pressure against Edwards syndrome cells, at least in vitro (110). No specific genes on chromosome 18 have been implicated in Edwards syndrome–specific abnormalities.

#### 4.3.3. Trisomy 13 (Patau syndrome).

Patau syndrome is marked by severe developmental abnormalities that often lead to early mortality. Among the general effects of aneuploidy mentioned above are cardiac defects, neurological disorders, and craniofacial abnormalities. The main specific features of Patau syndrome include feeding difficulties, microcephaly, holoprosencephaly, coloboma (a missing part in the eye), deafness, olfactory bulb agenesis, capillary hemangiomas, long hyperconvex nails, undescended testes, orofacial clefts, hydrocephalus, cleft lip and palate, renal anomalies, and polydactyly (207). Trisomy 13 impacts tissue-specific gene expression during the differentiation of embryoid bodies. Significant changes, particularly in brain-specific genes, have been observed in Patau syndrome–derived hESCs compared with diploid controls (23). No specific genes on chromosome 13 have been implicated in Patau syndrome–specific abnormalities.

#### 4.3.4. Sex chromosome aneuploidies.

Sex chromosome aneuploidies have been reviewed elsewhere (174), and here we summarize two examples: monosomy X (Turner syndrome) and gain of chromosome X in the presence of a Y chromosome (Klinefelter syndrome). As mentioned above, aneuploidies involving the X chromosome are less detrimental than autosomal aneuploidy due to X inactivation. However, approximately 15–20% of genes on the X chromosome escape X inactivation, and these genes remain active on both X chromosomes in females (15). As expected, the phenotypes of X chromosome aneuploidies, including Turner syndrome and Klinefelter syndrome, have been attributed to haploinsufficiency of genes that evade X inactivation. A key example is the *SHOX* gene located on Xp22.33, which encodes a transcription factor involved in bone development that normally escapes X inactivation (42). Decreased and increased *SHOX* dosages have been implicated in decreased and increased stature in Turner syndrome and Klinefelter syndrome, respectively (202). Interestingly, *SHOX* deletions or mutations also lead to Léri–Weill dyschondrosteosis, which shares skeletal features with Turner syndrome (42, 145). Other skeletal abnormalities, osteoporosis, cardiovascular abnormalities, gonadal dysgenesis, and infertility are common in Turner syndrome and Klinefelter syndrome (174).

## 5. FUTURE DIRECTIONS

### 5.1. The Aneuploidy-in-Cancer Paradox Through the Lens of Human Genetics

For a long time, there has been considerable debate about whether aneuploidy drives or inhibits cancer, particularly in the context of solid tumors, where it is highly prevalent. This topic has been extensively reviewed elsewhere (205); here, we briefly discuss its relevance at the intersection between cancer and congenital aneuploidy. In fact, one way to understand and resolve this apparent paradox is through the lens of human genetics. As described above, individuals with DS have a decreased risk of most solid tumors, a phenotype attributed to the triplosensitivity of specific genes on chromosome 21, such as *ETS2*, *RCAN1*, and *DYRK1A*. In human solid tumors, chromosome 21 is rarely gained. A similar analysis of cancer risk cannot be performed for other congenital autosomal aneuploidies, such as Patau syndrome and Edwards syndrome, due to the extremely short life expectancies of individuals with these syndromes. However, there is a syndrome in humans called mosaic variegated aneuploidy, caused by mutations in genes involved in chromosome segregation (e.g., *BUB1B*, *CEP57*, or *TRIP13*), which is characterized by CIN and mosaic aneuploidy. Patients with mosaic variegated aneuploidy syndrome have a high risk of developing several types of solid tumors, including Wilms tumor and rhabdomyosarcoma (60, 83). Therefore, from the perspective of human genetics, it can be argued that CIN and aneuploidy are generally protumorigenic, with the notable exception of specific chromosomal aneuploidies that lead to the increased dosage of tumor suppressor genes.

### 5.2. Ongoing Challenges and Future Directions

A key aspect of aneuploidy that makes it challenging to model and understand is that it involves a large number of genes—often hundreds, or even thousands. This complexity introduces significant technical and conceptual difficulties in dissecting aneuploidy. Understanding the consequences of a specific aneuploidy requires experimentally modeling the aneuploidy, defining its phenotypes, and determining which genes on the altered chromosome contribute to the observed phenotype and how they do so.

Technically, generating and examining aneuploid cells with sufficient karyotypic diversity is a significant challenge. However, recent advancements in genetic tools, such as KaryoCreate and ReDACT, present promising solutions to address this challenge (17, 27, 31, 56, 65, 187, 198, 200). In the future, the field will need to expand the use of these methods beyond cancer cells and immortalized cells, extending them to primary cells, including tissue stem cells, hESCs, iPSCs, and organoids. This expansion will enable us to significantly broaden our repertoire of available model systems, allowing us to address aneuploidy in the context of a 3D structure with ongoing cell-to-cell interactions and differentiation processes. Additionally, since each phenotype is likely influenced by multiple genes on the aneuploid chromosome, genetic tools are needed to simultaneously knock down or overexpress several genes in order to demonstrate that their combined gene dosage alterations are responsible for the observed phenotype. While this is feasible to do for a few genes (e.g., using multiple single guide RNA vectors) (1), it remains challenging to do for many genes simultaneously. However, recent advances in synthetic biology have made it possible to insert large genetic elements into the human or mouse genome, which will facilitate these efforts in the future (228).

One challenge when modeling aneuploidy in vivo in animal models is the lack of synteny between the mouse or rat genome and the human one. These models are still imperfect, as the DS mouse models do not recapitulate 100% of the human genes on chromosome 21 (94). Recent advances in human artificial chromosomes (HACs) and synthetic biology may help overcome this hurdle, leading to better models for both human cancer and congenital aneuploidy. On the one hand, recent developments in synthetic biology have enabled the insertion of synthetic genomic cassettes of up to 200 kb into the genome in a single step (228), allowing for the creation of new mouse models. On the other hand, HAC technology has made significant progress in overcoming the challenge of HAC multimerization, enabling the development of single HACs that can replicate and be transmitted stably as single copies in mammalian cells (58). Together, these tools will allow building better in vivo models of congenital and cancer trisomies.

Dissecting the molecular mechanism leading from an aneuploid genome to specific phenotypes is also challenging. For example, identifying the genes on the altered chromosome that drive the observed phenotypes is not trivial, as normally multiple genes collectively contribute. This complexity is further compounded by the fact that each gene has numerous downstream effects and interacts with other genes in the genome, both in *cis* (on the same chromosome) and in *trans* (on different chromosomes). The field needs to move beyond the traditional one gene–one phenotype relationship. Recent developments in computational methods, particularly those focused on cell signaling and gene-to-gene interactions, will be instrumental in achieving this goal. Machine learning has greatly advanced the study of complex genetic interactions within cells. It can aid in inferring gene regulatory networks from gene expression data and analyzing single-cell RNA sequencing to identify cell states and trajectories (77). A recent computational meta-analysis of subchromosomal copy number alteration data for almost 1 million genomes across 54 diseases unveiled 3,635 high-confidence dosage-sensitive genes contributing to disease phenotypes (44). Most dosage-sensitive genes are bidirectional, being both haploinsufficient and triplosensitive. These genes are characterized by evolutionary conservation and mutational constraint. The combination of experimental and computational tools—applied to both experimental systems and patient samples—will enable us to better understand the intricate gene interactions and cellular processes underlying aneuploidy.

Even if we cannot fully understand aneuploidy, we can begin to target it for medical purposes, a strategy that is already being explored, particularly in cancer therapy. There are two main approaches to targeting cancer-associated aneuploidy. The first involves developing therapies that target the general, nonspecific effects of aneuploidy, such as various types of cellular stress. One example is the use of inhibitors of KIF18A, a kinesin motor protein crucial for controlling chromosome alignment during mitosis (43). Highly aneuploid tumors are sensitive to disruptions in mitotic processes because they rely on precise chromosome alignment to maintain their abnormal karyotype. Inhibiting KIF18A exacerbates this instability by disrupting chromosome alignment and segregation, putting additional stress on these already compromised cells and leading to cell death. The second approach focuses on targeting chromosome-specific effects of aneuploidy. A clinically successful example is the drug lenalidomide, which is effective in multiple myeloma patients with a chromosome 5q deletion (112). Lenalidomide inhibits CSNK1A1, encoded by a haploinsufficient gene on chromosome 5q. Additionally, recent research has shown that cells with a 1q gain are more sensitive to treatment with 3-deazauridine, a synthetic nucleoside analog that inhibits DNA and RNA synthesis. Its metabolism is accelerated in cells with a 1q gain, as the rate-limiting step is its phosphorylation by UCK2, which is encoded by a gene located on chromosome 1q (65).

Thanks to novel experimental and computational approaches, we are beginning to uncover the molecular mechanisms underlying the hallmarks of aneuploidy, both in cancer and in congenital syndromes. The field is poised for significant advancements in the coming years and decades. These breakthroughs not only will enhance our fundamental understanding of aneuploidy but also will pave the way for improved therapeutic and preventative strategies for both cancer and congenital diseases.

## Figures and Tables

**Table 1:** Frequencies and main causes of aneuploidy in human cancer and development

Condition	Frequency	Main causes
Cancer	90% of solid tumors and 50% of hematopoietic cancers are aneuploid (187, 212, 226).	Incorrect kinetochore–microtubule attachments Supernumerary centrosomes DNA replication stress Mutation in cohesin and telomere crisis (109)
Oocyte maturation	More than 20% of eggs in women in their 20s and early 30s (84) and more than 50% of eggs from women aged 35 years and above (35) are aneuploid.	Unstable spindles, which are common in human oocytes (lower levels of KIFC1) (175) Errors in spindle attachment due to unpaired sister kinetochores and fragmented kinetochores (230, 231) Less stringent spindle assembly checkpoint in a large cytoplasm (101, 103) Decreased levels of cohesin (cohesion exhaustion) in aged oocytes (53, 113) Spindle-associated actin disruption (54)
Embryo development	73% of human embryos resulting from in vitro fertilization contain aneuploid cells (123, 203).	Abnormal mitotic spindle geometry (172) Anaphase lagging and mitotic nondisjunction (due to incorrect kinetochore–microtubule attachments) and chromosome demolition (123, 201) Premature cell division, errors in cytokinesis, cell fusion, and chromosome breakage (123)

**Table 2:** Chromosome-specific consequences of aneuploidy in cancer

Chr. gain/loss	Models	Categories	Selective advantages	Potential drivers	Refs.
1q gain	Elimination of 1q gain from cancer cell lines by ReDACT; lymphoma cell lines with 1q gain	Engineered (ReDACT); existing aneuploid cell lines	Blocks p53 signaling	MDM4	65, 85
1q gain	MPS1i-treated human mammary epithelial cells and long culture	Evolution (long culture)	Enhances NOTCH-induced proliferation	NCSTN	210
3p loss	CRISPR-mediated 3p deletion in human lung epithelial cells	Engineered (CRISPR)	No positive effect on proliferation	Not applicable	187
4p loss	scRNA sequencing of basal breast cancer tumors with 4p loss and 4p normal clusters, as well as normal and cancer cells with 4p loss	Heterogeneous cancer cells; existing aneuploid cell lines	Enhances proliferation	C4orf19	100
5q loss	Genetically engineered mouse models of breast cancer with Trp53 deletion; breast cancer mouse model with spontaneous loss of a region syntenic to human 5q33.2–35.3	Aneuploidy after TP53 deletion	Enhances high-glycolysis signaling and increases metastatic potential	Unknown; KIBRA	98, 127
6p loss	RPE1 cells resistant to MPS1i after long-term culture	Evolution (reversine)	Limits CIN by decreasing APC/C activity and extending mitotic durations	MAD2L1BP	2
7 gain	Human colonic epithelial cells and long culture under serum-free conditions (spontaneous 7 gain)	Evolution (serum-free)	Provides growth advantages in serum-free condition	Unknown	120
8 gain	Trisomy 8 fibroblast cell lines with EWS–FLI1 fusion; Ewing sarcoma samples with or without 8 gain	Existing aneuploid cell lines	Mitigates EWS–FLI1-induced replication stress	RAD21	180
8 gain	Trisomy 8 fibroblast	Engineered (MMCT)	Loses contact inhibition, enables regrowth after senescence	Unknown	136
8q gain	MPS1i-treated human mammary epithelial cells and long culture	Evolution (long culture)	Causes MYC-induced enhanced proliferation	MYC	210
8p loss	Immortalized lung epithelial cells with engineered 8p loss	Engineered (CRISPR)	Decreases cell death	WRN	169
8p loss	TALEN-engineered 8p loss in MCF10A cells; CRISPR-mediated deletion of a 33-Mb region of 8p in human liver cancers	Engineered (TALEN/CRISPR)	Triggers cell invasiveness, protects cells from hypoxic stress, and causes resistance to statin treatment	Collective of MSRA, NAT1, PPP2CB, and DLC1	31, 86
9p loss	Engineered mouse chromosome syntenic to human 9p21.3 deletion in pancreatic ductal epithelial cells	Engineered (MACHETE)	Promotes immune evasion, metastasis, and immunotherapy resistance	IFN cluster	17
10p loss	SAC-inhibited RPE1 cells and culture with drugs	Evolution (paclitaxel)	Causes resistance to paclitaxel	Unknown	118
11 gain	SAC-inhibited RPE1 cells and culture with drugs	Evolution (taxol)	Causes resistance to taxol	Unknown	89
11q loss	Engineered 11q deletion in neuroblastoma SK-N-SH cells	Engineered (CRISPR)	Increases clonogenic capacity	Unknown	56
13q gain	Isogenic system of colorectal cancer cells with or without 13 gain	Engineered (MMCT)	Enhances proliferation	KLF5	93
13q loss	Multiple cell lines resistant to reversine after long culture	Evolution (reversine)	Limits CIN by decreasing APC/C activity and extending mitotic durations	CDC16	2
17p loss	Cre-LoxP-mediated heterozygous deletion of mouse chromosome syntenic to human 17p13.1	Engineered (Cre-LoxP)	Promotes lymphoma and leukemia development	Cooperation of Eif5a and Alox15b with Trp53	116
18q loss	CRISPR-mediated 18q loss in human colonic epithelial cells	Engineered (KaryoCreate)	Causes resistance to TGF-β signaling	Unknown	27
Mouse 15 gain	Pretumoral T cell populations and lymphomas from a CIN mouse model (Cdc20^AAA/–^)	Evolution (in vivo)	Promotes thymic lymphomas	Myc, Rad21	199

Abbreviations: CIN, chromosomal instability; IFN, interferon; KaryoCreate, Karyotype CRISPR-Engineered Aneuploidy Technology; MACHETE, molecular alteration of chromosomes with engineered tandem elements; MMCT, microcell-mediated chromosome transfer; MPS1i, MPS1 inhibition; ReDACT, Restoring Disomy in Aneuploid Cells Using CRISPR Targeting; SAC, spindle assembly checkpoint; scRNA, single-cell RNA; TALEN, transcription activator–like effector nuclease.

**Table 3:** Chromosome-specific consequences of congenital aneuploidy

Chr. gain/loss	Models	Pathological relevance	Phenotypes	Potential drivers	Refs.
21 gain	Human DS iPSCs and in vitro differentiation into the neural crest	Craniofacial dysmorphology	Neural crest differentiation of human DS IPSCs showed normal proliferation/apoptosis but impaired migration, resulting in fewer postmigratory cranial neural crest cells in DS compared with controls.	*CXADR*	114
21 gain	DS fibroblasts	General phenotypes	DS fibroblasts exhibited two to four times higher apoptosis (spontaneous and induced) compared with matched controls.	*ETS2*	220
21 gain	MMCT-mediated DS mouse model; Ts65Dn DS mouse model	Leukemia	Trisomy 21 enhances production of hemogenic endothelial cells, hematopoietic stem cell precursors, and immature progenitors, increasing colony-forming potential, and induces macrocytosis and myeloproliferative disease with thrombocytosis, megakaryocyte hyperplasia, dysplasia, and, myelofibrosis.	*RUNX1, Erg*, exclude *Aml1/Runx1*	48, 96, 137
21 gain	Cells with and without trisomy 21; transcriptome analysis with shRNA screening	Autoimmune disorders	Trisomy 21 activates IFN response in multiple cell types. JAK1 and TYK2 kinases suppress trisomy 21 fibroblast proliferation, and JAK inhibitors rescue this defect.	Four IFN receptor genes on chromosome 21; *JAK1*, *TYK2*	182, 211
21 gain	Human DS iPSCs and in vitro and in vivo differentiation; Ts65Dn mouse model with different copies of DYRK1A	Neurological abnormalities	DS iPSCs form teratomas in mice; trisomy 21 causes a proliferation deficit and increased apoptosis in differentiated neural progenitors as well as reduced dendritic and synaptic development/activity in differentiated neurons. Dyrk1A normalization improved brain function and restored hippocampal cell proliferation/differentiation and synaptic markers.	*DYRK1A* (likely through REST/NRSF,WNT, and NOTCH signaling); WNT signaling	61, 64, 80, 214
21 gain	Human DS iPSCs; genetic normalization of *Olig1*/*Olig2* gene dosage in Ts65Dn mice	Neurological abnormalities	Human DS iPSCs overproduce OLIG2^+^ ventral forebrain progenitors, leading to excess GABAergic interneurons in cerebral organoids and impaired recognition memory in neuronal chimeric mice. Correcting *Olig1*/*Olig2* gene dosage reduced inhibitory neurons and improved the excitatory/inhibitory balance in Ts65Dn mice.	*OLIG2*	32, 224
21 gain	Ts65Dn and Ts1Cje mouse models of DS; ~1,200 organoids with and without 21 gain	Neurological abnormalities	Elevated *App* gene dosage reduces NGF retrograde transport, leading to degeneration of basal forebrain cholinergic neurons. Subtle neurodevelopmental phenotypes are present in DS brain development around the second trimester.	*App*; increase in secreted Ab40 peptide levels	45, 158
21 gain	Ts1Cje mice	Neurological abnormalities	NMDA rapidly triggered DSCAM protein synthesis in dendrites of normal neurons but not in Ts1Cje neurons.	*DSCAM*	5
21 gain	*APC*^*Min*^-mediated tumor formation in DS mice; injection of tumor cells into DS mice	Solid tumor development	Trisomy 21 reduces APCMin intestinal tumors in Ts65Dn and Ts1Rhr mice but increases them in Ms1Rhr mice, and suppresses tumor formation of transplanted cancer cell lines in Ts65Dn and Tc1 mice with repressed VEGF-mediated angiogenesis.	*ETS2, DSCR1, DYRK1A, ADAMTS1, ERG*	12, 149, 183

Abbreviations: DS, Down syndrome; IFN, interferon; iPSC, induced pluripotent stem cell; MMCT, microcell-mediated chromosome transfer; shRNA, short hairpin RNA.

## CHROMOSOMAL GAINS, LOSSES, AND WHOLE-GENOME DUPLICATION

Aneuploidy manifests as gains or losses of chromosomes. Chromosomal gains, including trisomy (the presence of an extra chromosome), result in increased gene dosage. Conversely, chromosomal losses, such as monosomy (the absence of one chromosome from a pair), lead to reduced gene dosage. Aneuploidy can occur at various scales: locally (affecting small chromosomal regions, also commonly referred to as focal aneuploidy), at the arm level (involving entire chromosome arms), or encompassing whole chromosomes (22). The mechanisms causing aneuploidy differ by scale. Focal aneuploidy often results from DNA replication errors, breakage–fusion–bridge cycles, or chromothripsis (105). Arm-level aneuploidy typically occurs through DNA breaks at the centromere, missegregation of dicentric chromosomes, or breakage of anaphase bridges. Whole-chromosome aneuploidy arises primarily from mitotic errors such as merotelic attachments, cohesion defects, or spindle assembly checkpoint dysfunction, leading to chromosome missegregation (13, 66). Whole-genome doubling can result from cytokinesis failure, mitotic slippage, or cell fusion (105). Unlike aneuploidy, whole-genome doubling results in a proportional increase of all chromosomes, meaning that the relative dosage of each chromosome or gene remains unchanged compared with the diploid state. In tumorigenesis, whole-genome doubling often serves as a precursor to more complex aneuploidies, providing a buffer against deleterious mutations and promoting CIN. These aberrations significantly impact cellular function via both shared and distinct mechanisms.

## CHROMOSOMAL INSTABILITY AND ANEUPLOIDY

Aneuploidy arises through CIN during mitosis or meiosis. While often discussed together, CIN and aneuploidy are distinct: CIN is the ongoing rate of acquiring chromosomal alterations, while aneuploidy is the static state of an abnormal chromosome number. CIN generates aneuploid cells, and aneuploidy can promote CIN in certain contexts (e.g., through increasing replication stress). However, it is crucial to note that aneuploid cells are not necessarily chromosomally unstable (6, 204). For instance, the human colorectal carcinoma cell line HCT116 and cells from individuals with Down syndrome maintain stable aneuploidy without ongoing CIN. This review focuses primarily on aneuploidy; for a comprehensive review of CIN, we direct readers to Reference 66.

From a technological standpoint, aneuploidy can be assessed via metaphase spreading, fluorescence in situ hybridization (FISH), or whole-genome sequencing, whereas directly measuring CIN from a single time point is challenging. Cell population copy number heterogeneity, enhanced by single-cell sequencing, approximates recent CIN. Developing robust CIN quantification methods remains a challenge with potential for research and clinical advancements (6, 91). CIN and aneuploidy can induce both shared and unique consequences. Thus, carefully distinguishing CIN and aneuploidy and their unique effects is of paramount importance. To this end, two strategies can be employed: identifying CIN-specific effects that disappear when CIN halts but aneuploidy persists (14) and using aneuploid models without CIN to isolate aneuploidy-specific effects.

## Abbreviations

Aneuploidy: an abnormal number of chromosomes in a cell due to chromosome gains or losses
Whole-genome doubling: duplication of the entire genome, resulting in polyploidy
Congenital aneuploidy: a condition in which an individual is born with an abnormal number of chromosomes
Proteotoxic stress: cellular stress caused by the accumulation of misfolded or damaged proteins, overwhelming protein quality control mechanisms
Chromosomal instability (CIN): the occurrence of an unstable genome with an increased rate of gaining or losing a chromosome or of accumulating chromosomal structural aberrations
Kinetochore–microtubule attachments: connections between chromosomes and spindle fibers during cell division, which are essential for proper chromosome segregation
Kinetochore: the protein structure that attaches chromosomes to spindle fibers during cell division
Spindle assembly checkpoint (SAC): a cell cycle control mechanism that ensures proper chromosome attachment before anaphase onset
Centrosome amplification: the presence of extra centrosomes in a cell, often leading to multipolar spindles and CIN
Telomere dysfunction: the impairment of protective chromosome end structures, potentially causing genomic instability and cellular senescence
Haploinsufficiency: a condition where a single functional gene copy is insufficient to maintain normal cellular function
Unfolded protein response: a cellular stress response activated by accumulation of misfolded proteins in the endoplasmic reticulum
Karyotypic heterogeneity: cell-to-cell variability in aneuploid profiles in cell populations
Immune evasion: a set of mechanisms employed by cancer cells to avoid detection and destruction by the immune system
Triplosensitivity: a condition where a single gain of a functional gene copy is sufficient to change normal cellular function
X inactivation: a process in female mammals where oneX chromosomes is randomly silenced, equalizing X-linked gene expression with males
Mosaic aneuploidy: a condition where only a subset of cells in a tissue or body carry aneuploidy, i.e., chromosome gain or loss
Holoprosencephaly: a condition that alters the development of a baby’s brain
